# KDM2A and KDM3B as Potential Targets for the Rescue of F508del-CFTR

**DOI:** 10.3390/ijms23179612

**Published:** 2022-08-25

**Authors:** Claudio D’Amore, Christian Borgo, Valentina Bosello Travain, Mauro Salvi

**Affiliations:** 1 Department of Biomedical Sciences, University of Padova, 35031 Padova, Italy; 2 Department of Molecular Medicine, University of Padova, 35031 Padova, Italy

**Keywords:** genetic disease, therapeutic target, demethylases, protein degradation, posttranslational modifications

## Abstract

Cystic fibrosis (CF) is caused by mutations in the gene encoding of the cystic fibrosis transmembrane conductance regulator (CFTR), an anion-selective plasma membrane channel that mainly regulates chloride transport in a variety of epithelia. More than 2000 mutations, most of which presumed to be disease-relevant, have been identified in the CFTR gene. The single CFTR mutation F508del (deletion of phenylalanine in position 508) is present in about 90% of global CF patients in at least one allele. F508del is responsible for the defective folding and processing of CFTR, failing to traffic to the plasma membrane and undergoing premature degradation via the ubiquitin–proteasome system. CFTR is subjected to different post-translational modifications (PTMs), and the possibility to modulate these PTMs has been suggested as a potential therapeutic strategy for the functional recovery of the disease-associated mutants. Recently, the PTM mapping of CFTR has identified some lysine residues that may undergo methylation or ubiquitination, suggesting a competition between these two PTMs. Our work hypothesis moves from the idea that favors methylation over ubiquitination, e.g., inhibiting demethylation could be a successful strategy for preventing the premature degradation of unstable CFTR mutants. Here, by using a siRNA library against all the human demethylases, we identified the enzymes whose downregulation increases F508del-CFTR stability and channel function. Our results show that KDM2A and KDM3B downregulation increases the stability of F508del-CFTR and boosts the functional rescue of the channel induced by CFTR correctors.

## 1. Introduction

Cystic fibrosis is a genetic disease caused by hundreds of different mutations of *CFTR*, a gene encoding the CFTR protein, a chloride/HCO_3_^−^ channel of the plasma membrane [1,2]. The most common mutant, F508del-CFTR, is characterized by: (i) a severe defect in trafficking leading to its degradation at the ER level, (ii) a gating defect, and (iii) instability at the plasma membrane when corrected [3,4]. The current therapy for this type of mutation (Trikafta) is based on the correction of trafficking defects (mediated by VX-445 and VX-661 drugs) combined with a potentiator (VX-770) [5,6,7]. CFTR is subjected to different post-translational modifications (PTMs), including phosphorylation, SUMOylation, ubiquitination, acetylation, and methylation, and the control of their turnover was proposed as a potential therapeutic intervention [8,9]. However, the identification of PTMs whose regulation could be critical for the functional recovery of CFTR mutants is still lacking. Recently, the existence of a PTM hotspot, the so-called “PTM code”, was hypothesized, with a role in CFTR maturation [10]. Indeed, the increased phosphorylation of four residues (T421, S422, S427, and S434) and the methylation of K442 were found in wild-type CFTR or corrected F508del-CFTR, while the lack of phosphorylation at these specific sites, and the concurrent ubiquitination of K420 and K442 were found in noncorrected F508del-CFTR and a misfolded N1303K-CFTR variant [10]. The phosphorylation of T421, S422, and S427 was attributed to CK2 [10], a constitutively active protein kinase [11] that is considered to be a potential therapeutical target for treating different human diseases [12], and whose role in F508del-CFTR maturation was recently investigated [13]. However, with a mutational approach, we recently showed that this PTM code is dispensable for the functional recovery of F508del-CFTR, suggesting that the regulation of PTMs at these sites would not be relevant for a potential therapeutic intervention [14].

The recent PTM mapping of CFTR shows that there are several methylation sites besides the two present in the putative PTM code whose function is still to be highlighted [10]. Protein methylation was recently recognized as an important PTM that is not limited to histone proteins, but widely diffused and taking part in the regulation of different signal transduction pathways [15,16]. We, therefore, wonder if protein methylation could play a general role in CFTR maturation, and if the targeting of the enzymes responsible for the turnover of this PTM could be a valuable strategy for the functional recovery of F508del-CFTR. Some lysine residues that undergo methylation could, indeed, also be ubiquitinated. Proteasomal degradation via polyubiquitination is the main degradative route of the channel [17,18], and we and others very recently demonstrated that preventing protein ubiquitination at the beginning of the enzymatic cascade could be an effective therapy to improve the correction of F508del-CFTR and other misfolded mutants [19,20]. This observation leads us to hypothesize that preserving methylation could be a way to prevent F508del-CFTR ubiquitination and degradation, resulting in the increased stability of the channel.

## 2. Results and Discussion

Recently, a new PTM mapping of CFTR showed that the protein could be modified by methylation on different lysines; some of these residues could also be modified by ubiquitination, suggesting a competition between the two PTMs [10]. Therefore, we reasoned that preserving the methylation of F508del-CFTR at these sites, e.g., by downregulating specific demethylates, should prevent their ubiquitination and possibly channel degradation.

**Figure 1:**
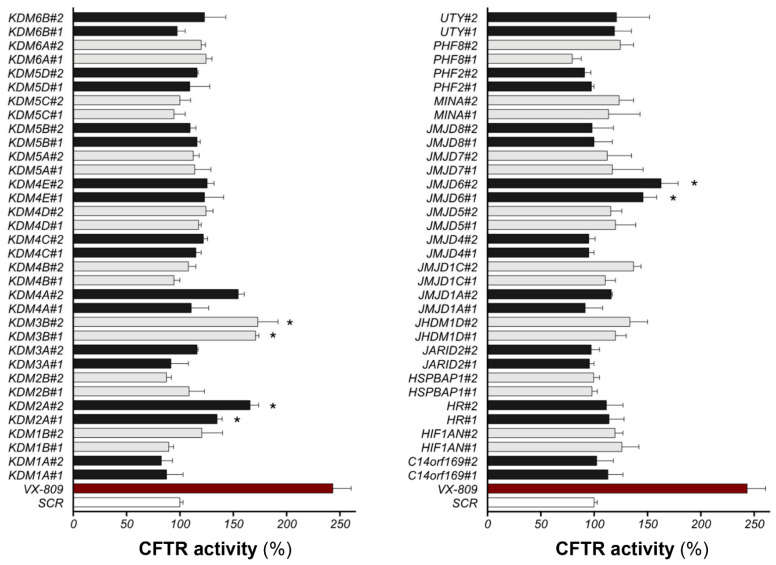
SiRNA screening against all human demethylases. CFBE41o- stably overexpressing F508del-CFTR and HS-YFP were transfected for 48 h with two different siRNAs for each target or with scrambled siRNAs. The panel indicates the CFTR activity as a percentage of control (SCR) (scrambled-siRNAs + DMSO) (mean  ±  SD, *n*  =  8; * *p*  <  0.05 vs. SCR). The extent of rescue obtained with 3 μM VX-809 is indicated by a red bar for comparison.

To identify the demethylases involved in F508del-CFTR stability, we employed a siRNA library individually targeting all human demethylases. The potential rescue of F508del-CFTR after demethylase downregulation was quantified by assaying channel activity using a halide-sensitive *YFP* assay. Two different siRNAs for each target were transfected in CFBE41o- cells stably overexpressing F508del-CFTR and the halide-sensitive yellow fluorescent protein (HS-YFP). After 48 h, F508del-CFTR activity at the plasma membrane was assessed by measuring the rate of HS-YFP quenching caused by iodide influx into cells. As a positive control, we treated cells with the VX-809 corrector. The YFP technique is a powerful method to measure CFTR channel function, and the reliability of the technique was confirmed here with the downregulation of CFTR, which completely abolished YFP quenching (Figure S1). The results of the siRNA library screening against all the human demethylases are displayed in Figure 1. For each individually silenced gene target, we evaluated the resulting CFTR activity, normalized to the activity measured in control cells treated with nontargeting siRNAs. The functional screening identified three protein targets, namely, JMJD6, KDM2A, and KDM3B: the downregulation of these targets by both two different siRNAs led to significant F508del-CFTR functional rescue based on the assessment of HS-YFP quenching.

**Figure 2:**
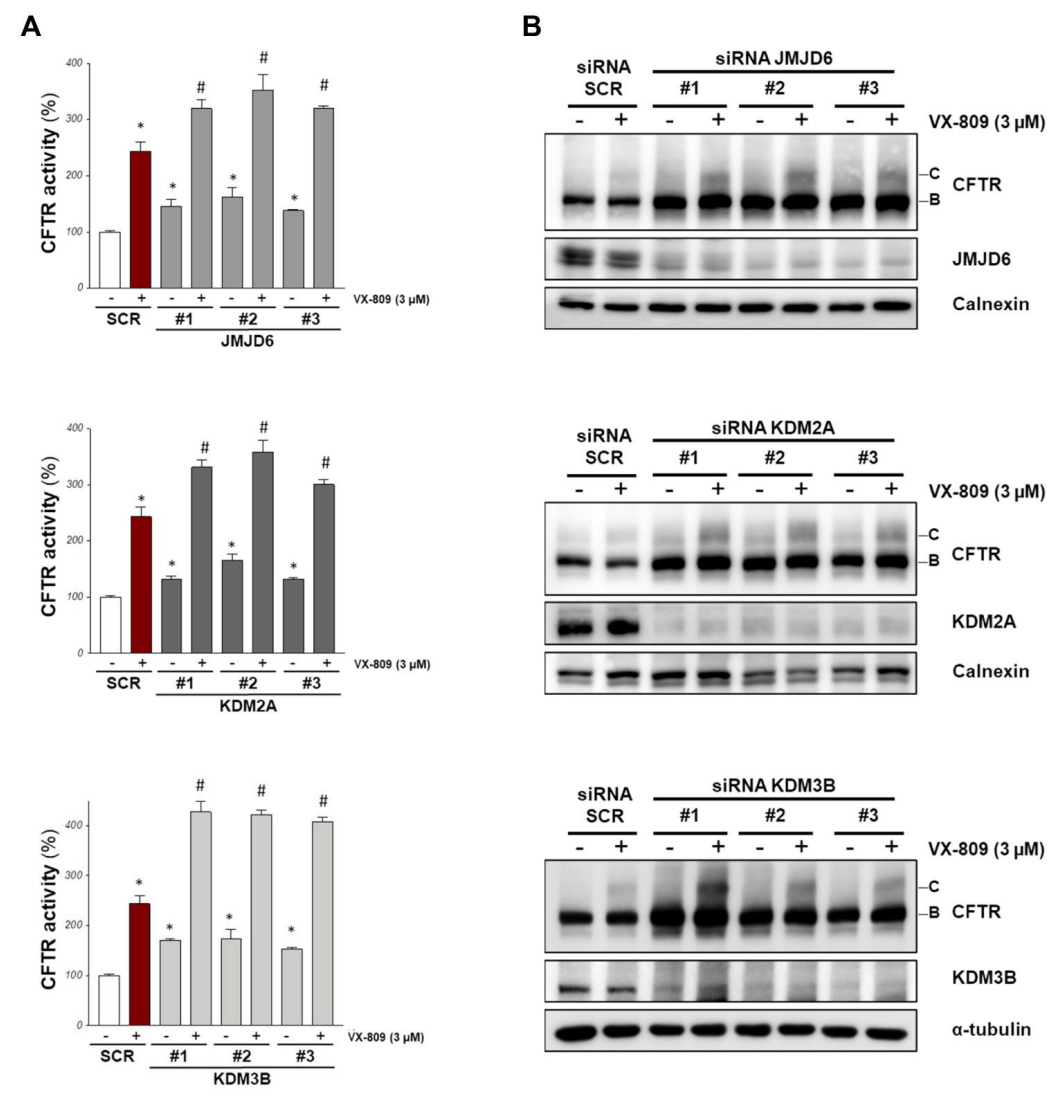
Validation of the most promising demethylase targets. CFBE41o- stably overexpressing F508del-CFTR and HS-YFP were transfected with three different siRNA for each indicated target or with scrambled siRNAs. After 24 h cells were treated with 3 μM VX-809 or with vehicle (DMSO) for further 24 h. (**A**) The graphs indicate the CFTR activity as a percentage of control (SCR, scrambled-siRNAs + DMSO) (means ± SD values, *n* = 8; * *p* < 0.05 vs. SCR, # *p* < 0.05 vs. SCR + VX-809 for cells treated with VX-809). (**B**) The panel shows the western blotting of cell lysates treated as in A to analyze the downregulation of the siRNA targets and the F508del-CFTR expression. Calnexin or α-tubulin were used as loading control. Densitometry analysis of immunostained bands was performed in Figure S2.

We proceeded to a validation step using a third different siRNA against the three selected demethylases, confirming the knockdown of the targets with specific antibodies. All the three siRNA treatments against JMJD6, KDM2A, and KDM3B resulted in an increase in F508del-CFTR activity (Figure 2A) and an increment of the channel band B amount assessed via Western blotting (Figure 2B and Figure S2).

Lastly, we also evaluated whether a combined treatment of RNA silencing with a CFTR corrector such as VX-809 results in a stronger effect. We transfected CFBE41o- cells with siRNAs against the selected demethylases, and the following day, we treated silenced cells with either a vehicle (DMSO) or the VX-809 corrector. After an additional 24 h, F508del-CFTR activity in the plasma membrane was measured using the YFP assay, and the channel expression was evaluated with Western blotting. Our results show that the siRNA treatments boosted the effect of VX-809, as judged by assaying the functional rescue of F508de-CFTR (Figure 2A) and the expression of its mature form (band C; Figure 2B).

The increased expression of F508del-CFTR after demethylase downregulation could be attributed to the gene transcription induction or an increase in channel stability.

**Figure 3:**
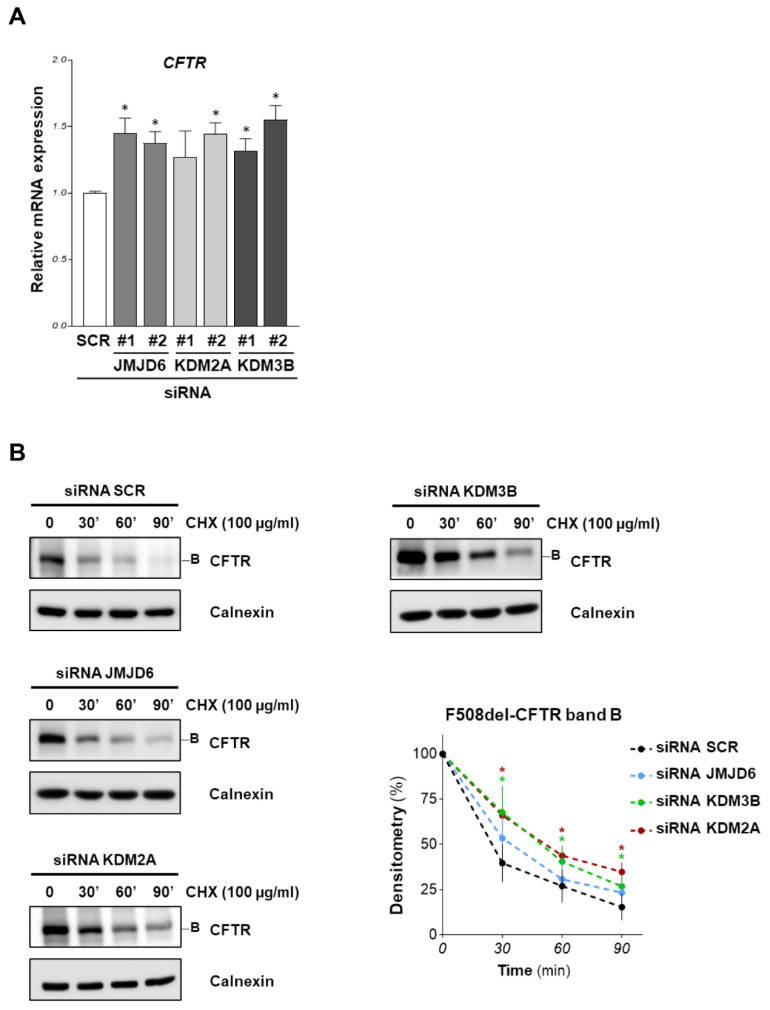
Transcriptional and stability analysis of F508del-CFTR after demethylases downregulation. (**A**) *CFTR* mRNA levels determined by quantitative real-time PCR in F508del-CFTR expressing CFBE41o- cells were transfected with two different siRNAs for each target (JMJD6, KDM2A, KDM3B) for 48 h. *CFTR* mRNA expression was normalized to 18S RNA and reported relative to its expression in control (SCR) cells that was arbitrarily set to 1 (means ± SD values, *n* = 5). (**B**) F508del-CFTR overexpressing CFBE41o- cells were transfected with siRNA targeting JMJD6, KDM2A, KDM3B or with scrambled siRNAs for 48 h. Subsequently, protein synthesis was inhibited by adding 100 µg/mL cycloheximide (CHX) and cells were harvested after 30, 60 and 90 min. Protein lysates were analyzed by western blot with an anti-CFTR antibody. Calnexin was used as a loading control. The lower-right graph represents the densitometric quantification of the immunostained bands of F508del-CFTR band B normalized by the value at time  =  0 (mean  ±  SD, *n*  =  4; * *p*  <  0.05 vs. the value of the same time-point of SCR).

A qRT-PCR was performed to assay the effects of siRNAs targeting KDM2A, KDM3B, and JMJD6 on *CFTR* expression in CFBE41o- cells. Figure 3 shows that JMJD6 and KDM3B downregulation by both siRNAs induces a small but significant increase in *CFTR* mRNA expression. F508del-CFTR protein stability was evaluated by treating F508del-CFTR overexpressing CFBE41o- cells with the specific siRNAs for 48 h. Subsequently, protein synthesis was inhibited by adding cycloheximide (CHX) to the cells; then, cells were harvested at 0, 30, 60 and 90 min after CHX treatment. F508del-CFTR band B level was quantified with Western blotting at each time point. Figure 3B shows that F508del-CFTR band B stability increased after KDM2A and KDM3B downregulation, but not after JMJD6 downregulation.

In summary, our results show that KDM2A and KDM3B demethylases could be considered to be new molecular targets to increase F508del-CFTR stability and improve the efficacy of CFTR correctors.

However, it remains to be clarified if the band B stabilization induced by the downregulation of the two lysine demethylases is due to a direct effect on the methylation of F508del-CFTR or an indirect effect on methylation of other proteins controlling F508del-CFTR stability. The main role of protein methylation, which is relatively small compared to other modifications and with a limited effect on charge distributions, seems to be the regulation of protein–protein interactions [16,21]. Therefore, protein methylation signaling could regulate one or more molecular complexes with a role in CFTR processing, directly targeting the channel or other proteins involved in its proteostasis.

**Figure 4:**
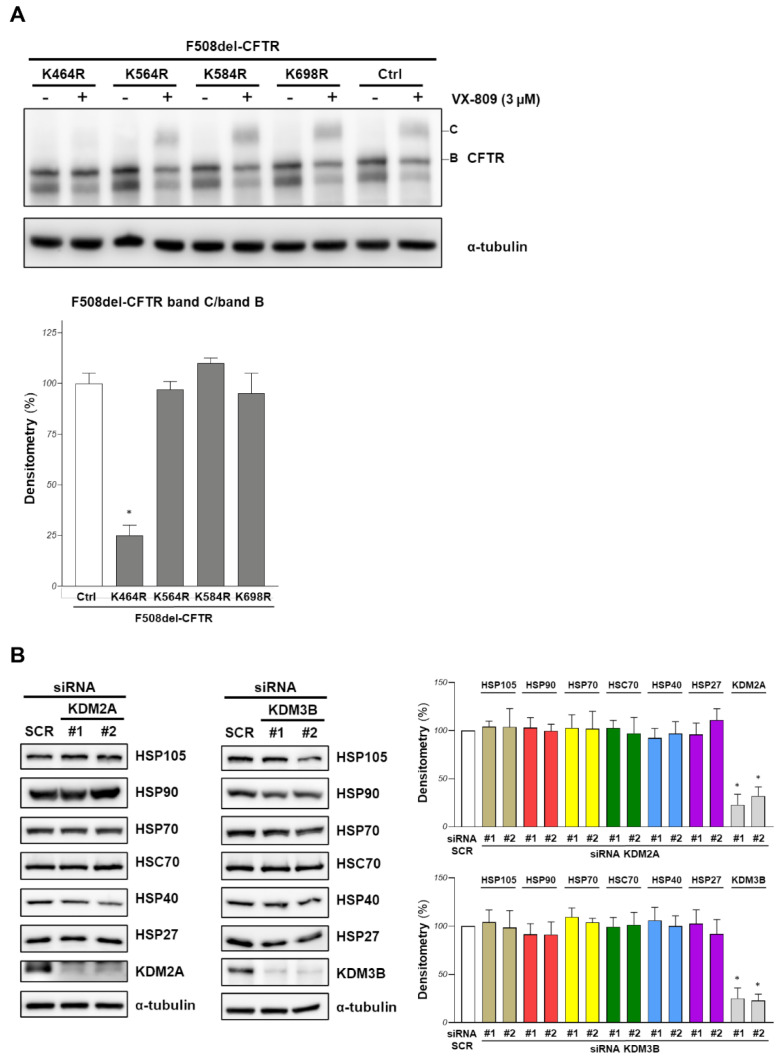
Mutational analysis of CFTR methylated lysine and effect of demethylases downregulation on chaperone machinery. (**A**) CFBE41o- cells were transiently transfected with F508del-CFTR, or other derivative mutants as indicated. After 24 h, cells were treated with 3 μM VX-809 and the amounts of F508del-CFTR band B and band C were assessed by western blotting. α-tubulin was used as loading control (upper panel). In the lower panel, the graphs show the ratio of F508del-CFTR band C/band B for the cells treated with VX-809, obtained by the densitometric quantification of the immunostained band C normalized by band B expression, and expressed as a percentage of the control cells (Ctrl) (means ± SD values, *n* = 4; * *p* < 0.05 vs. Ctrl). (**B**) F508del-CFTR expressing CFBE41o- cells were transfected with siRNA (#1 and #2 guides) targeting KDM2A or KDM3B or with a scrambled (SCR) siRNA for 48 h. In the left and central panels, the expression of the indicated chaperones was analysed by western blotting. α-tubulin was used as loading control. In the right panel, the graphs report the densitometric quantification of the immunostained bands of the experiment detailed in the left and central panels normalized by α-tubulin expression, and expressed as a percentage of the control cells (SCR). (Means ± SD values, *n* = 3; * *p* < 0.05 vs. SCR).

To shed some light on this point, we treated F508del-CFTR overexpressing CFBE41o- cells with siRNAs targeting KDM2A or KDM3B or scrambled siRNAs for 48 h, and we immunoprecipitated F508del-CFTR to assess its methylation state with Western blotting and an anti-methylated lysine antibody, but the results were inconclusive due to the low efficiency of these antibodies (data not shown). Therefore, we conducted the mutational analysis of all lysine residues that had been identified as methylated in CFTR. We previously showed that the mutations of K420 and K442 (K/R) do not change the F508del-CFTR band B/C expression [14]. Here, we assayed the effect of K464, K564, K584 and K698 mutations in arginine (K/R). F508del-CFTR and the K/R mutants were transfected in CFBE41o- cells. After 24 h of transfection, we treated the cells with the VX-809 corrector, and band B and C amounts were assayed with Western blotting. Figure 4A shows that none of the lysines that could be modified by methylation increased band B or C amounts, showing that the modification of at least these residues plays no role in F508del-CFTR stability and maturation. The K464R mutant (a lysine located in NBD1 that is critically involved in ATP binding) cannot be corrected by VX-809 despite a similar band B expression to that of the other mutants (Figure 4A). This result confirms a previous observation on K464A mutation [22] that showed trafficking defects when inserted in both F508del and wild-type CFTR, suggesting that ATP binding at NBD1 is required for channel maturation.

The chaperone machinery is involved in the recognition of misfolded proteins, favoring their folding or addressing the protein to degradation. Targeting this pathway is often proposed as a mechanism to increase F508del-CFTR protein stability or maturation [23,24]. Therefore, we evaluated if the effect of KDM2A or KDM3B silencing on the channel rescue could be due, at least partially, to a perturbation of the chaperone machinery involved in F508del-CFTR recognition and degradation. Figure 4B shows that no alteration in protein expression was observed for the main chaperones (HSP105, HSP90, HSP70, HSC70, HSP40, HSP27) that are involved in F508del-CFTR stability and processing.

To sum up, even if further work is necessary to detail the precise mechanism of action, our results identify KDM2A and KDM3B demethylases as two new molecular targets to control F508del-CFTR proteostasis and to boosts the effect of CFTR correctors.

## 3. Materials and Methods

### 3.1. Materials

VX-809 was purchased from MedChemExpress (Monmouth Junction, NJ, USA). The anti-CFTR antibody (#596) was obtained from the Cystic Fibrosis Foundation Therapeutics (Bethesda, MD, USA). The anti-α-tubulin (T5168) antibody was purchased from Merck (Darmstadt, Germany). The anti-KDM3B (GTX116198) antibody was from GeneTex (Irvine, CA, USA). Anti-KDM2A (FBXL11) (NB100-74602) antibodies were from Novus Biological (Centennial, CO, USA). Anti-JMJD6 (sc-28348), anti-calnexin (sc-46669), anti-Hsp105 (sc-74550), anti-Hsp90 (sc-7947), anti-Hsp70 (sc-32239), anti-Hsc70 (sc-7298), anti-Hsp40 (sc-398766), anti-Hsp27 (sc-13132), and anti-ubiquitin (sc-8017) antibodies were from Santa Cruz Biotechnology (Dallas, TX, USA). Antimouse and antirabbit HRP-conjugated secondary antibodies were from PerkinElmer (Waltham, MA, USA). siRNA oligos were obtained from Thermo Fisher Scientific (Waltham, MA, USA).

### 3.2. Cell Culture

CFBE41o- expressing an endogenous level of F508del-CFTR or stably overexpressing F508del-CFTR and YFP-H148Q/I152L (a kind gift from Prof. L.J.V. Galietta, TIGEM, Italy) was cultured in a MEM cell culture medium supplemented with 10% FBS, 2 mM L-glutamine, and antibiotics, and maintained at 37 °C in a humidified atmosphere of 5% CO_2_.

### 3.3. Cell lysis and Western Blotting

Cells were washed twice with PBS and harvested with ice-cold lysis buffer containing 50 mM Tris-HCl (pH 7.5), 150 mM NaCl, and 1% NP-40 (*v*/*v*), supplemented with protease inhibitor cocktail (Merck), and phosphatase inhibitor cocktails 2 and 3 (Merck). Cell lysates were centrifuged at 10,000× *g* for 15 min at 4 °C, and protein concentration in the supernatant was determined with the Bradford method. Then, 20 µg of total protein extracts were loaded onto SDS-PAGE, blotted on Immobilon-P membranes (Merck), and processed with Western blot with the indicated antibodies. Immunostained bands were detected with chemiluminescence on ImageQuant LAS 500 (GE Healthcare Life Sciences, Hatfield, UK), and quantified with Carestream Molecular Imaging software v.5.0.5.31 (Carestream, Rochester, NY, USA).

### 3.4. Cell transfection and RNA Interference

Plasmid transfections in CFBE41o- cells were performed as in [14]. For RNA interference, CFBE41o-overexpressing F508del-CFTR were transiently transfected for 48 h with 5 nM siRNA or scrambled siRNA (Silencer Select siRNAs, Thermo Fisher Scientific; the list of siRNA used in this study is shown in Table S1) with RNAiMAX (Thermo Fisher Scientific), according to the manufacturer’s instructions.

### 3.5. CFTR Mutagenesis

CFTR mutants were obtained with the PCR method as described in [14]. All plasmids were sequenced to confirm mutagenesis.

### 3.6. HS-YFP Assay

CFTR activity was measured using CFBE41o- cells stably expressing both F508del-CFTR and the halide-sensitive YFP (H148Q/I152L) according to [25] with minor modifications. Briefly, cells were seeded on 96-well black microplates and let to adhere for 24 h. Cells were treated with the indicated compounds for a further 18 h, with eight replicate wells for each condition. On the day of the experiment, cells were washed with PBS (137 mM NaCl, 2.7 mM KCl, 8.1 mM Na_2_HPO_4_, 1.5 mM KH_2_PO_4_, 1 mM CaCl_2_, and 0.5 mM MgCl_2_), and incubated with 20 µM forskolin and 50 µM genistein in PBS to fully stimulate F508del-CFTR for 25 min at 37 °C. Then, plates were transferred to an EnVision plate reader (PerkinElmer) to determine CFTR activity: YFP fluorescence (Ex:485/14; Em:535/30) was continuously measured for 15 s, 2 s before and 13 s after the injection of an iodide-enriched solution (185 mM NaI, 2.7 mM KCl, 8.1 mM Na_2_HPO_4_, 1.5 mM KH_2_PO_4_, 1 mM CaCl_2_ and 0.5 mM MgCl_2_); the final I^-^ concentration was 100 mM.

To determine I^−^influx rate and CFTR activity, background fluorescence was subtracted, and fluorescence was normalized to the initial value; the final 12 s of the data recording for each well was fitted with an exponential function to calculate the initial slope (dF/dt).

### 3.7. qRT-PCR

CFBE41o- cells stably expressing F508del-CFTR were seeded at 3 × 10^5^ cells/well in a 6-well plate, left to adhere for 24 h, and treated with the control or demethylases targeting siRNAs for 48 h. Total mRNA was isolated with a Total RNA Purification kit (Norgen, Biotek Corp., Thorold, ON, Canada) and reverse-transcribed using SuperScript III reverse transcriptase (ThermoFisher). For quantitative RT-PCR, 5 ng of template was dissolved in a 20 µL solution containing forward and reverse primers (200 nM each) and 10 µL of SensiFAST SYBR No-ROX Mix, 2× (Bioline, Cincinnati, OH, USA). All the reactions were performed in triplicate on a Real-Time PCR Cycler Rotor-Gene 3000 (Corbett Research Ltd., Crawley, UK); the thermal cycling conditions were as follows: 95 °C for 3 min, 40 cycles of 95 °C for 10 s, 58 °C for 20 s, and 72 °C for 30 s. The relative mRNA expression was calculated and expressed as 2^−ΔΔCt^. Primers were as follows: h18S AAACGGCTACCACATCCAAG and CCTCCAATGGATCCTCGTTA; hCFTR CTGGGCTAGGGAGAATGATG and GCCTTCCGAGTCAGTTTCAG.

### 3.8. CFTR Stability

F508del-CFTR overexpressing CFBE41o- cells were treated as indicated. Subsequently, protein synthesis was inhibited by adding 100 µg/mL cycloheximide (CHX) to the culture medium; then, cells were harvested at different time points as shown in Figure 3B. Cell lysates were subjected to SDS-PAGE, followed by Western blotting to evaluate F508del-CFTR expression.

### 3.9. Statistical Analysis

Results are presented as mean ± SD; all experiments were repeated at least three times. A comparative analysis of the differences between the two groups was performed using Student’s *t*-test (two-tailed), and comparisons of the differences between the groups were performed using one-way ANOVA with post hoc Tukey’s test. Differences were considered to be significant with a *p*-value < 0.05.

## Data Availability

The data are available from the corresponding authors upon reasonable request.

## Supplementary Materials

The following supporting information can be downloaded at: https://www.mdpi.com/article/10.3390/ijms23179612/s1.
