# Supplementary material for: KDM2A and KDM3B as Potential Targets for the Rescue of F508del-CFTR
Source: Int J Mol Sci. 2022 Aug 25;23(17):9612. doi: 10.3390/ijms23179612 (PMC9455907; doi:10.3390/ijms23179612)
Supplement: Supplementary file 1 [file ijms-23-09612-s001.zip › Supplementary figures-author-re.pdf]

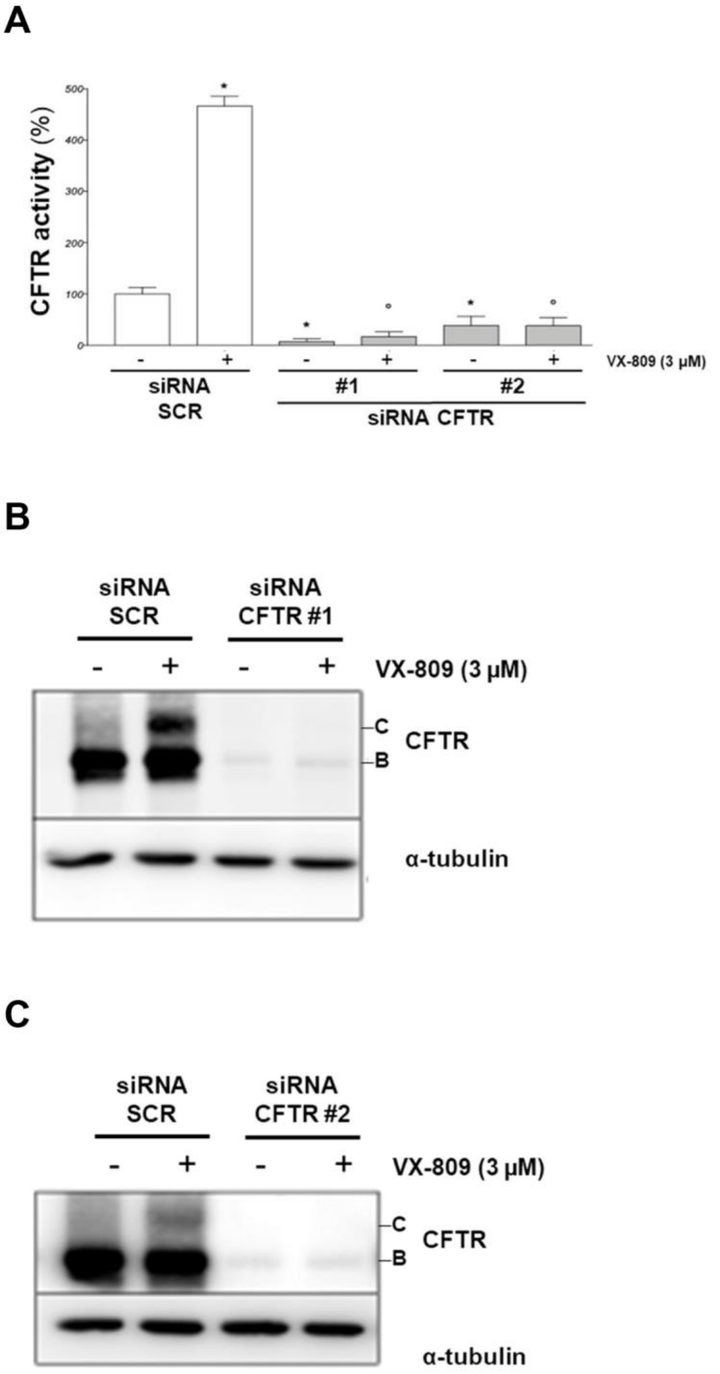

Fig. S1. Validation of YFP-assay

CFBE41o- cells overexpressing F508del-CFTR were transfected with two different siRNA targeting CFTR or with a scrambled siRNA. After 24 h cells were treated with 3 μM VX-809 or with the vehicle (DMSO) for a further 24 h. (A) The panel indicates the CFTR activity as a percentage of control (SCR) (means ± SD values, $n = 8$; $^*p < 0.05$ *vs* SCR). (B) and (C) show the western blotting of cell lysates treated as in A and analyzed with anti-CFTR antibody. α-tubulin was used as loading control.

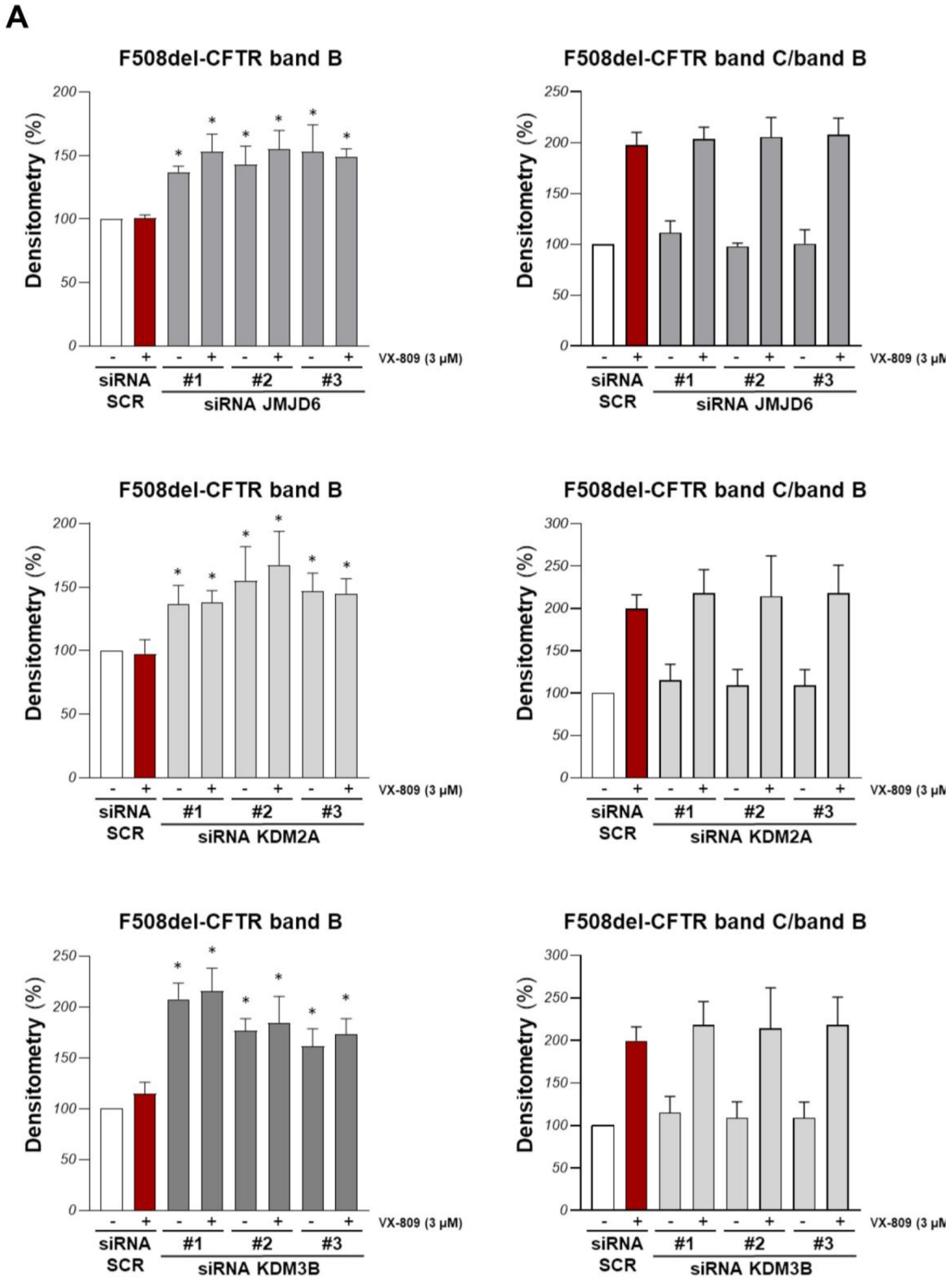

Fig. S2. Densitometric analysis of western blot experiment in Fig. 2B

The graphs report the densitometric quantification of immunostained F508del-CFTR band B (lef panel) or F508del-CFTR band C/band B ratio (righ panel) relative to the experiment described in Fig 2B. In the case of F508del-CFTR band B quantification, the densitometric quantification of th immunostained band B was normalized by α-tubulin expression, and expressed as a percentage of th control cells (SCR) (means ± SD values, n=4; *$p < 0.05$ *vs* SCR). In the case of F508del-CFTR ban C/band B quantification, the densitometric value of the immunostained band C was normalized b band B expression, and expressed as a percentage of the control cells (SCR) (means ± SD values n=4; *$p < 0.05$ *vs* SCR for cells treated with DMSO, #$p < 0.05$ *vs* SCR+VX-809 for cells treated wit VX-809).
